# Therapeutic Peptide Amphiphile as a Drug Carrier with ATP-Triggered Release for Synergistic Effect, Improved Therapeutic Index, and Penetration of 3D Cancer Cell Spheroids

**DOI:** 10.3390/ijms19092773

**Published:** 2018-09-14

**Authors:** Sheng Lu, Feng Zhao, Qiuxin Zhang, P. Chen

**Affiliations:** 1 Department of Chemical Engineering and Waterloo Institute for Nanotechnology, University of Waterloo, Waterloo, ON N2L 3G1, Canada; shanels825@gmail.com; 2 Faculty of Science, University of Waterloo, Waterloo, ON N2L 3G1, Canada; zhaofeng87806698@163.com (F.Z.); q344zhang@edu.uwaterloo.ca (Q.Z.); 3 College of Nano Science and Technology, Soochow University, Suzhou 215123, Jiangsu Province, China

**Keywords:** peptide amphiphile, membrane lytic, synergistic effect, cancer selectivity

## Abstract

Despite the great progress in the field of drug delivery systems for cancer treatment over the last decade, many challenges still lie ahead, such as low drug loading, deep penetration of tumors, side effects, and the development of drug resistance. A class of cationic membrane lytic peptides has shown potential as an anticancer agent by inducing cancer cell death via membrane disruption; meanwhile, their intrinsic selectivity renders them as having low cytotoxicity towards noncancerous cells. Here, we report the use of a cationic peptide amphiphile (PA), named PAH6, to load doxorubicin (Dox) that is intercalated in an ATP-binding aptamer-incorporated DNA scaffold. The PA contains a cationic lytic sequence, (KLAKLAK)_2_, a polyhistidine segment for the “proton sponge” effect, and a hydrophobic alkyl tail to drive the self-assembly. Dox-loaded DNA was found to form a spherical nanocomplex (NC) with PAH6 with particle sizes below 100 nm at various ratios. Since the carrier PAH6 is also a therapeutic agent, the drug loadings of the NC reached up to ~86% within the ratios we tested, and Dox was released from the NC in an ATP-rich environment. In vitro studies indicate that the presence of PAH6 could permeabilize cell membranes and kill cells through fast membrane disruption and depolarization of mitochondrial membranes. The cytotoxicity tests were conducted using A549 nonsmall cell lung cancer cells and NIH-3T3 fibroblast cells. PAH6 showed selectivity towards A549 cells. Significantly, the Dox-DNA/PAH6 NC exhibited a synergistic effect against A549 cells, with the IC50 decreased up to ~90% for Dox and ~69% for PAH6 when compared to the IC50 values of the two components, respectively. Furthermore, the selectivity of PAH6 conferred to the complex an improved therapeutic index between A549 and NIH-3T3 cells. A 3D-cultured A549 spheroid model was adopted to test the capability of Dox-DNA/PAH6 for tumor penetration. The PAH6 or Dox-DNA/PAH6 complex was found to break the spheroids into pieces, while Dox-treated spheroids maintained their shapes. In summary, this work provides a new strategy for constructing nanomedicines using therapeutic agents to meet the features required by anticancer treatment.

## 1. Introduction

The development of anticancer nanomedicine is aiming for improved pharmacokinetics and therapeutic efficacy of the drugs with lowered side effects. Over the past decades, researchers have engineered a large number of nanoparticles made from various materials (organic, inorganic, natural, etc.) with diverse physicochemical properties (for example, geometry, surface chemistry, stiffness, size, etc.), and have accumulated substantial understanding on how the physicochemical properties affect the drug delivery process and tumor accumulation [1,2,3,4]. In particular, nanoparticles with size ranging between 10 and 100 nm are found to passively accumulate in tumor sites through enhanced permeability and retention effect [4,5], and reduced nanoparticle size can facilitate tumor penetration [6,7]; fibrous nanostructures are reported to accumulate in tumors better than their spherical counterparts [8]; and spherical nanoparticles show advantages in cellular uptake [9]. Additional functions, such as stimulus-triggered drug release [10,11,12,13], enhanced cell membrane translocation [14,15,16], and tuned cancer cell selectivity [11,17], are further engineered to equip nanoparticles for better therapeutic efficacy and reduced side effects. Numerous biomarkers have also been identified and targeting molecules are mounted on the surface of nanoparticles for improved tumor accumulation [18,19].

Despite these advances, few nanomedicines have been successful in clinical trials. One of the major reasons for the failure is insufficient tumor penetration of the nanomedicines, resulting from the increased interstitial fluid pressure due to the leaky vasculature and poor lymphatic drainage of tumor tissue and hindered diffusion caused by condensed cell packing and extracellular matrix [20,21,22]. The majority of the aforementioned nanomedicine designs have targeted the anticancer mechanisms at a cellular or molecular level, neglecting the distribution of the nanomedicine within the tumor tissues. A study even found that surface modification of a nanoparticle with a targeting ligand would hinder the tumor penetration [23]. Moreover, the drug-loading ability of nanomedicines limits the concentration of drugs penetrating into tumor tissues [24]. Therefore, nanomedicines with high penetrating capability and drug-loading capacity need to be achieved to overcome these barriers.

Peptides have received increased interest in biomedical research and development due to their functional versatility and selectivity, as well as their relative safeness. For instance, cell-penetrating peptides or protein transduction domains are a class of functional peptides widely used in drug/gene delivery systems, facilitating the internalization and tumor penetration of the cargo [25,26,27]. Some of these peptides are also found to promote the endo/lysosomal escape of their cargo [27,28,29] and direct them to localize at cell nuclei [30,31]. Another class of cationic amphiphilic peptides, deriving from naturally occurring proteins, have shown antimicrobial and/or anticancer activities, mainly via physically disrupting the phospholipid membranes or triggering apoptotic pathways [32,33,34,35]. Moreover, these peptides possess intrinsic selectivity towards cancerous cells and bacteria, rendering them promising as therapeutic agents.

Cationic polymers have been shown to have the potential to overcome the physical barriers that prevent their tumor penetration [36,37,38]. Sharing the similar membrane disruption mechanisms, we hypothesize that cationic anticancer peptides would also be able to penetrate tumor tissues. Previously, our group reported the use of cationic anticancer peptides to deliver an anticancer drug, ellipticine, and demonstrated that the systems can significantly enhance the therapeutic efficacy towards cancerous cells, but were barely enhanced towards normal cells [11,17]. However, the interactions between the drug and peptides rely on hydrophobic interactions, which are not stable and are nonspecific. Ran et al. reported the use of an ATP-aptamer incorporated double-stranded DNA scaffold to load doxorubicin (Dox) and release it in response to the high intracellular concentration of ATP [12,39]. In this work, we designed a cationic peptide amphiphile (PA), PAH6, as an anticancer agent with the ability to penetrate the tumor. The PAH6 contains an anticancer sequence, (KLAKLAK)_2_ [35,40], connected by a glycine linker with a polyhistidine moiety for extra positive charges under an acidic environment. A palmitic tail is conjugated at the N-terminus to provide hydrophobic driving force for nanoparticle formation. We adopted the reported ATP-aptamer incorporated DNA scaffold to load Dox. The Dox-loaded ATP-aptamer incorporated DNA scaffold can interact with PAH6 through electrostatic attractions to form a spherical nanocomplex (NC) and release Dox in an ATP-triggered manner. We investigated the cell killing mechanisms of the NC and its cancer selectivity and synergistic effect on monolayer-cultured A549 lung cancer cells, HCT116 colorectal cancer cells, and NIH-3T3 fibroblast cells. The ability of the NC to penetrate tumors was tested on a 3D-cultured A549 cell spheroid model. In summary, this work provides a new route of constructing nanomedicines using therapeutic agents to meet the features required by anticancer treatment. 

## 2. Results

### 2.1. Dox Loading and Release from DNA Scaffold

**Figure 1:**
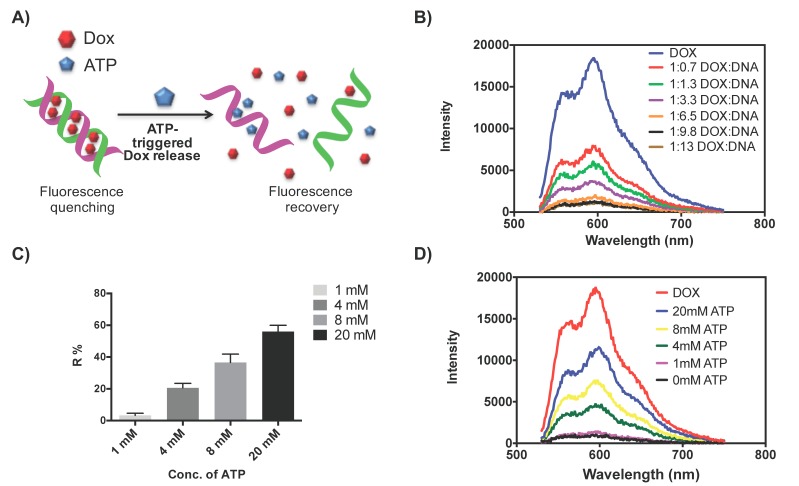
(**A**) Schematic illustration of the ATP-triggered release of Dox from the ATP-binding aptamer incorporated DNA scaffold. (**B**) The fluorescence spectra of Dox (1 µg/mL) with increasing mass ratios of the DNA scaffold. (**C**) The percentage of Dox fluorescence recovery from the Dox-DNA in the presence of different concentrations of ATP (1, 4, 8, 20 mM). Error bars represent SD (*n* = 3). (**D**) the fluorescence spectra of Dox-DNA (1 µg/mL) at the mass ratio of Dox to DNA of 1:10 in the presence of different concentrations of ATP (1, 4, 8, 20 mM).

We first prepared the Dox-loaded DNA scaffold by incubating Dox with the DNA scaffolding in an aqueous solution. The Dox molecules are supposed to intercalate into the GC-rich region of the DNA scaffold [41]. In the presence of ATP, the ATP-aptamer strand forms a stable tertiary structure with ATP molecules [42,43], resulting in the dissociation of the DNA scaffold and release of the intercalated Dox molecules (as shown in Figure 1A). When Dox molecules intercalate into the DNA scaffold, their proximity can initiate Föster resonance energy transfer, which causes the decrement of Dox fluorescence emission [12,44]. The correlation between the intercalation status of Dox molecules and their fluorescence intensity was used to monitor the Dox loading and release from the DNA scaffold. As shown in Figure 1B, the fluorescence emission of Dox was monitored at a fixed concentration and decreased with the increment of DNA concentration. A maximum fluorescence quenching was reached at a Dox/DNA mass ratio of ~1:9.8. This ratio is in line with previously reported results [12,39]. We chose the mass ratio of 1:10 for the following experiments. The ATP-triggered Dox release was then investigated with the presence of ATP at various concentrations (Figure 1C,D). As shown in Figure 1D, the fluorescence recovery of Dox was observed in an ATP concentration-dependent manner (up to 20 mM). The amount of released Dox was further estimated by the fluorescence recovery ratio ((*F_re_*-*F_q_*)/(*F_d_*-*F_q_*)), where *F_re_* represents the Dox fluorescence intensity in the presence of ATP, F_d_ represents the fluorescence intensity of Dox solution, and F_q_ represents the fluorescence intensity at the maximum quenching condition. As shown in Figure 1C, the percentages of recovered Dox fluorescence intensities were determined to be ~20%, ~36%, and ~56% at ATP concentrations of 4, 8, and 20 mM, respectively, much greater than that at the ATP concentration of 1 mM.

### 2.2. Characterization of the Dox-DNA/PAH6 Nanocomplex

**Figure 2:**
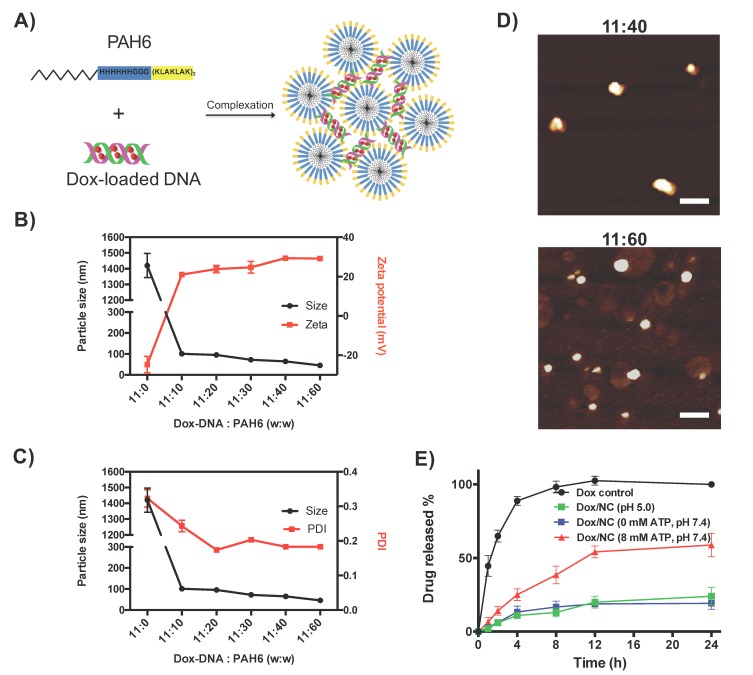
(**A**) Schematic illustration of the mechanism for the formation of the Dox-DNA/PAH6 NC. (**B**) The averaged particle size and zeta potential of the Dox-DNA/PAH6 NC at different mass ratios of Dox-DNA and PAH6. Error bars represent SD (*n* = 3). (**C**) The averaged particle size and polydispersity index (PDI) of the Dox-DNA/PAH6 NC at different mass ratios of PAH6 and Dox-DNA. Error bars represent SD (*n* = 3). (**D**) AFM images of the Dox-DNA/PAH6 NC at the mass ratios of Dox-DNA to PAH6 of 11:40 and 11:60, respectively. The scale bar indicates 100 nm. (**E**) The Dox release profile of the Dox-DNA/PAH6 NC in the presence of 8 mM ATP. Error bars represent SD (*n* = 5).

The Dox-loaded DNA scaffold (Dox-DNA, mass ratio 1:10) was then mixed with PAH6 to form the NC. According to the nature of the intermolecular interactions that participate in the process, the mechanism of the complexation between Dox-DNA and PAH6 was proposed as illustrated in Figure 2A. We found that PAH6 did not self-assemble into ordered nanostructures (Figure S1), which could result from the dominant electrostatic repulsion provided by the cationic amino acid residues. Neutralized by the negatively charged Dox-DNA, the attractive hydrophobic interactions and back-bond hydrogen bonding would drive the self-assembly of PAH6 to form micelles; the cationic micelles are linked by the Dox-DNA to generate the NC. The sizes and zeta potentials of the NC formed at different Dox-DNA/PAH6 mass ratios were characterized to find an optimal ratio. As determined by dynamic light scattering (DLS), the NC showed an average size distributed at ~100 nm with the Dox-DNA/PAH6 mass ratio of 11:10. With the increased ratio of PAH6, the size of the NC decreased to ~45 nm (Figure 2B). The polydispersity (PDI) of the NC was maintained at ~0.2 when the Dox-DNA/PAH6 mass ratio was above 11:20 (Figure 2C), demonstrating a relatively narrow size range. The zeta potential of the NC reached +30 mV above the Dox-DNA/PAH6 mass ratio of 11:30, demonstrating a good colloidal stability in aqueous solution. Considering that the smaller size of the NC allowed it to associate with more surface-exposed PAH6 that can contact cell membranes and improve the colloidal stability, the Dox-DNA/PAH6 mass ratios of 11:40 and 11:60 were adopted in the following studies. In these two cases, as both PAH6 and Dox are therapeutic agents, the drug loadings reach 80% and 86%, respectively.

Atomic force microscopy (AFM) was then used to investigate the nanostructures of Dox-DNA/PAH6. The AFM images showed that both Dox-DNA/PAH6 (11:40) and Dox-DNA/PAH6 (11:60) formed spherical nanoparticles and their size distributions were consistent with the DLS results (Figure 2D).

Next, we evaluated the ATP-triggered Dox release profile in the presence and absence of ATP using a dialysis tube. The Dox-DNA/PAH6 (11:40) NC exhibited a sustained Dox release when compared to the Dox control solution (Figure 2E). Furthermore, with the presence of 8 mM ATP, 38% of the intercalated Dox was released from the NC within the first 8 h and 58% was released up to 24 h; in contrast, without the presence of ATP, the Dox release was much slower, with only 20% of Dox released after 24 h (Figure 2E). We further investigated the effect of an acidic environment on the Dox release and found that the release of Dox was not accelerated at pH 5.0 (Figure 2E). Taken together, the results demonstrate the ATP-triggered Dox release characteristics of the Dox-DNA/PAH6 NC. 

### 2.3. Membrane Permeabilization and Mitochondrial Depolarization Induced by PAH6

**Figure 3:**
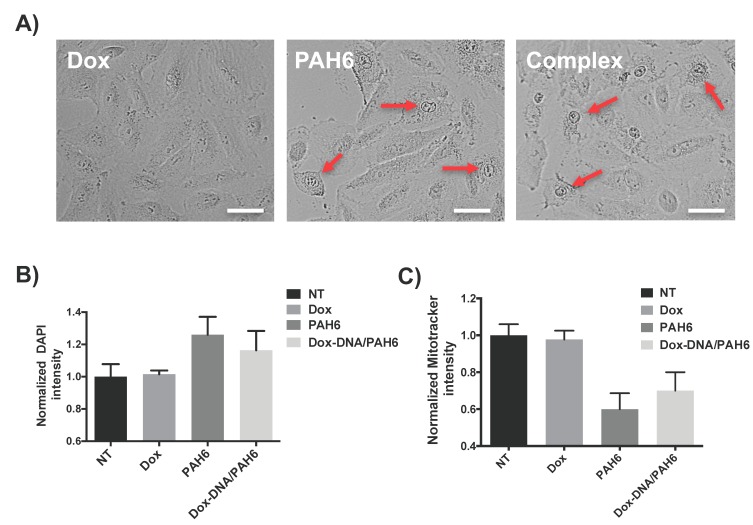
(**A**) Optical micrographs showing the morphologies of the A549 cells after 30 min incubation with Dox (2 µg/mL), PAH6 (120 µg/mL), and the Dox-DNA/PAH6 (11:60) NC at corresponding concentrations. The scale bar indicates 40 µm. Arrows indicate the cells undergoing necrotic death. (**B**) The normalized fluorescence intensity of DAPI permeating into the cells after 30 min incubation with Dox (1 µg/mL), PAH6 (60 µg/mL), and the Dox-DNA/PAH6 (11:60) NC at corresponding concentrations. The DAPI fluorescence intensity of the nontreated (NT) cells is set as 1 for intensity normalization. Error bars represent SD (*n* = 3). (**C**) The normalized fluorescence intensity of MitoTracker Red that binds to the mitochondrial membranes of the cells after 1 h incubation with Dox (1 µg/mL), PAH6 (60 µg/mL), and the Dox-DNA/PAH6 (11:60) NC at corresponding concentrations. The MitoTracker Red fluorescence intensity of the nontreated (NT) cells is set as 1 for intensity normalization. Error bars represent SD (*n* = 3).

The therapeutic components of the NC, Dox and PAH6, are supposed to induce cell death via different modes of action. The major cell killing mechanisms of Dox are well known, involving the intercalation into DNA and inhibition of topoisomerase-II-mediated DNA repair [45]. Meanwhile, the (KLAKLAK)_2_ peptide was reported to kill cells via membrane disruption and depolarization of mitochondrial membranes [35]. To investigate the cell killing mechanisms of the NC, A549 lung cancer cells were treated with Dox, PAH6, and Dox-DNA/PAH6 (11:60) NC. After 30 min of incubation, we observed the cell morphologies of the treated A549 cells using optical microscopy. The Dox-treated cells were found to be intact, while a proportion of the PAH6- and NC-treated cells exhibited a spread morphology with bright nuclei (Figure 3A), which suggests necrotic cell death induced by rapid cell membrane disruption. This also confirms that PAH6 still functions after forming the NC.

In previous studies, we proposed that the membrane disruptive ability of cationic anticancer peptides could enhance the permeability of the cell membrane in contact, facilitating the entry of small-molecule drugs and resulting in synergy [11,17]. After we confirmed the membrane disruptive ability of PAH6, the cell membrane permeabilization induced by PAH6 was evaluated by measuring the fluorescence intensity of DAPI permeated into A549 cells. As shown in Figure 3B, the normalized DAPI fluorescence intensity of the cells treated with PAH6 or Dox-DNA/PAH6 (11:60) NC presented a ~20% increase when compared to that of the nontreated cells. This suggests that PAH6 can enhance the permeability of cell membranes. The Dox-treated cells showed comparable DAPI intensity with the nontreated cells (Figure 3B), demonstrating that Dox barely influences the cell membrane permeability.

To investigate the mechanism of membrane depolarization, we further stained the mitochondrial membranes of the A549 cells with MitoTracker Red. The fluorescence intensity of the staining correlates with the membrane potential of mitochondria. The depolarization of mitochondrial membranes should be reflected by the reduction of the MitoTracker Red fluorescence intensity. As shown in Figure 3C, the normalized MitoTracker fluorescence intensity of PAH6- or Dox-DNA/PAH6 (11:60) NC-treated cells reduced ~40% and 30%, respectively, when compared to that of nontreated cells; meanwhile, the Dox-treated cells did not exhibit a reduction in the MitoTracker fluorescence intensity. In summary, PAH6 and Dox-DNA/PAH6 can increase cell permeability by disrupting cell membranes and depolarizing mitochondrial membranes.

### 2.4. Synergistic Effect and Selectivity of the Dox-DNA/PAH6 Nanocomplex

**Figure 4:**
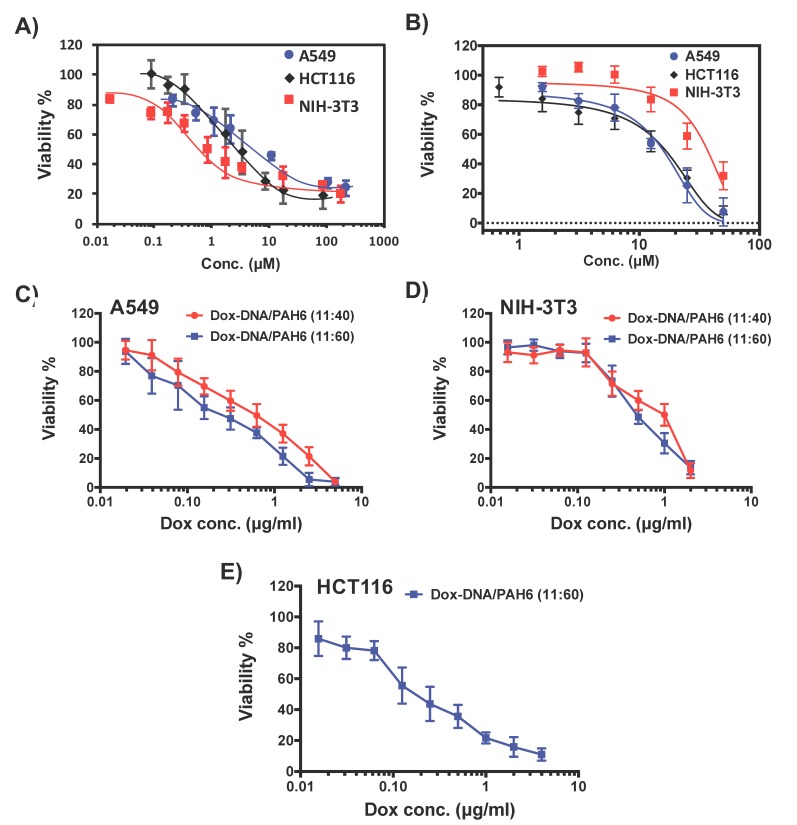
The in vitro cytotoxicity of (**A**) Dox, (**B**) PAH6, and (**C**–**E**) Dox-DNA/PAH6 at the mass ratios of Dox-DNA to PAH6 of 11:40 and 11:60 on A549, HCT116, or NIH-3T3 cells. Error bars represent SD (*n* = 5).

The in vitro cytotoxicity tests were conducted on A549 cells and NIH-3T3 fibroblast cells with 24 h of treatment. Cell counting kit-8 (CCK-8) was used to evaluate the viable cells. Dox was found to be more cytotoxic against NIH-3T3 cells than A549 cells (Figure 4A). The IC50 value of Dox was 3.8 + 0.4 µM (2.1 ± 0.2 µg/mL) on A549 cells and 0.86 ± 0.19 µM (0.47 ± 0.10 µg/mL) on NIH-3T3 cells. Meanwhile, PAH6 exhibited a selective activity towards A549 cells (Figure 4B). The IC50 of PAH6 was found to be 15.6 ± 1.2 µM (43.0 ± 3.3 µg/mL) on A549 cells and 36.8 ± 4.7 µM (101.4 ± 12.9 µg/mL) on NIH-3T3 cells.

In contrast, the NC presented significantly enhanced cytotoxicity against A549 cells, with an IC50 of 0.94 ± 0.24 μM (0.51 ± 0.13 µg/mL) Dox and 7.4 ± 1.9 μM (20.4 ± 5.2 µg/mL) PAH6 for Dox-DNA/PAH6 (11:40), and an IC50 of 0.42 ± 0.15 μM (0.23 ± 0.08 µg/mL) Dox and 5.0 ± 1.7 μM (13.8 ± 4.8 µg/mL) PAH6 for Dox-DNA/PAH6 (11:60), demonstrating the synergistic effect between PAH6 and Dox. More importantly, the enhanced cytotoxicity of the NC was not observed in NIH-3T3 cells (Figure 4D). The IC50 of the NC was 1.3 + 0.2 μM (0.70 ± 0.11 µg/mL) Dox and 10.2 ± 1.6 μM (28.0 ± 4.4 µg/mL) on NIH-3T3 cells for Dox-DNA/PAH6 (11:40), and 0.96 + 0.17 μM (0.52 ± 0.09 µg/mL) Dox and 11.3 ± 1.9 μM (31.2 ± 5.4 µg/mL) for Dox-DNA/PAH6 (11:60). The IC50 values of Dox in the NC were even higher than that of Dox itself. This might be due to the incomplete Dox release within 24 h. We further tested another cancerous cell line, HCT116 colorectal cancer cells, to confirm the selectivity and synergistic effect. It was found that Dox and PAH6 exhibited IC50 values of 1.89 ± 0.67 μM (1.03 ± 0.36 µg/mL) (Figure 4A) and 16.2 ± 1.5 µM (44.6 ± 4.1 µg/mL) (Figure 4B) on HCT116 cells, respectively. The IC50 of Dox-DNA/PAH6 (11:60) was determined to be 0.39 ± 0.09 μM (0.21 ± 0.05 µg/mL) Dox and 4.6 ± 1.7 μM (12.6 ± 4.8 µg/mL) (Figure 4E), which is comparable to the IC50 with A549 cells. Taken together, PAH6 and Dox in the NC can work synergistically on killing cancerous A549 and HCT116 cells, but not on noncancerous NIH-3T3 cells, significantly improving the therapeutic index (the determination of drug synergy is described in the Materials and Methods section). 

### 2.5. Anticancer Activity Against Lung Cancer Cell Spheroids

**Figure 5:**
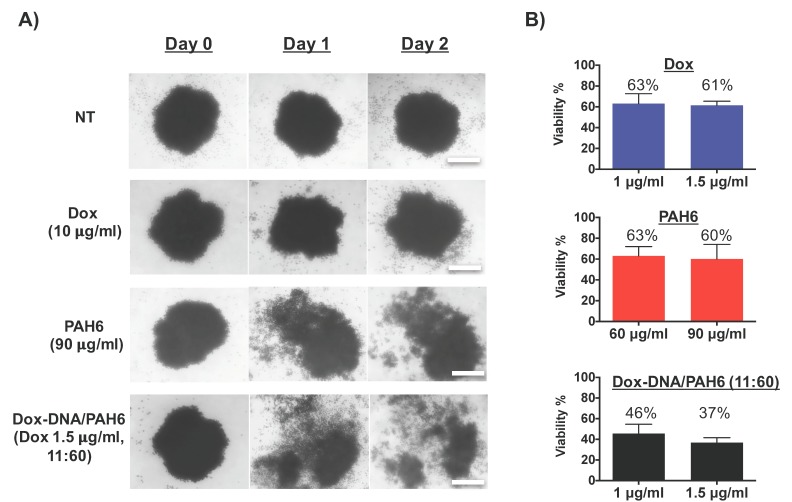
(**A**) Optical micrographs of 3D A549 spheroids treated with Dox, PAH6, and Dox-DNA/PAH6 NC, respectively. The scale bar indicates 260 µm. (**B**) The cytotoxicity of Dox, PAH6, and Dox-DNA/PAH6 NC against 3D A549 spheroids. Error bars represent SD (*n* = 5).

After cytotoxicity evaluation on monolayer-cultured cells, the anticancer activity of the Dox-DNA/PAH6 NC to combat A549 lung cancers was further tested on A549 spheroids. The spheroids were prepared by growing A549 cells in a pendant drop of culture media. The grown A549 spheroids were treated with Dox, PAH6, and Dox-DNA/PAH6 NC, respectively, and the morphologies of the spheroids were imaged. As shown in Figure 5A, the PAH6- and Dox-DNA/PAH6 (11:60)-treated spheroids were broken into pieces after 1 d of incubation, and the cells were eliminated further after 2 d of incubation. In contrast, Dox lacked the ability to penetrate the spheroids, and hence, only peeled off the A549 cells at the surface of the spheroids (Figure 5A). Even when the concentration of Dox was increased to 40 µg/mL, the treated spheroid still stayed as an entity (Figure S2). The anticancer activity tests were also performed on the 3D-cultured A549 spheroids (Figure 5B). Similar to the results obtained from monolayer-cultured A549 cells, Dox-DNA/PAH6 exhibited an enhanced therapeutic activity against the 3D spheroids compared to individual Dox or PAH6 treatments after 2 d of incubation. Both Dox and PAH6 killed ~40% of the A549 cells that formed the spheroids at the tested concentrations (1 µg/mL and 1.5 µg/mL for Dox; 60 µg/mL and 90 µg/mL for PAH6), whereas the Dox-DNA/PAH6 (11:60) NC killed 54% of the cells at the lower concentration and 63% at the higher concentration.

## 3. Discussion

In order to enhance the tumor penetrating ability and improve the therapeutic index of nanomedicines, we proposed to utilize the membrane disruptive ability of cationic PAs to achieve these goals. Therefore, we adopted a therapeutic PA, PAH6, to form NCs with a Dox-loaded DNA scaffold via electrostatic interactions. The DNA scaffold incorporated an ATP-aptamer strand, conferring to the NC the feature of triggered Dox release at the intracellular ATP level [12,46]. The PAH6 was found to induce fast membrane disruption and increase the membrane permeability. Utilizing this mechanism, the NC exhibited significantly enhanced therapeutic efficacy against A549 lung cancer cells, with the IC50 decreased by up to ~90% for Dox and ~69% for PAH6 when compared to the IC50 values of the two components respectively. The synergy could originate from the enhanced cellular uptake of Dox through the permeabilized cell membranes as we proposed previously [10,17], as well as the multiple killing mechanisms. Furthermore, the selectivity of PAH6 conferred to the NC the improved therapeutic index of Dox from 0.22 up to 2.26 between NIH-3T3 and A549 cells, and from 0.46 to 2.48 between NIH-3T3 and HCT116 cells, which are close to the PAH6 selectivity of 2.36. The reason could be that NIH-3T3 cell membranes were less permeabilized in contact with PAH6 than A549 cell membranes at the same concentrations, resulting in a barely synergistic effect on NIH-3T3 cells.

As we expected, PAH6 showed remarkable penetrating power on 3D-cultured A549 spheroids, conferring to the Dox-DNA/PAH6 NC improved anticancer ability. Although highly cationic polymers, such as polyethyleneimine (PEI), can also irritate cell membranes via electrostatic interactions and penetrate tumors [38], peptides hold several advantages involving intrinsic selectivity towards cancerous cells, safe degraded products, and flexibility in sequence design. The high drug-loading nanomedicines have gained attention because the extensive use of carrier materials may cause systemic toxicity and increase the burden of material clearance from the body [24]. Self-assembly of small-molecule drugs to form nanoparticles has been recently reported as a high drug-loading strategy [47]. Herein, we also provide a strategy of using therapeutic agents for constructing nanomedicines. Moreover, the multiple cell killing mechanisms of the NC might prevent the cancer cells from generating drug resistance, which could be another potential advantage of this strategy. 

Further development of this strategy for in vivo applications can involve several aspects: (1) modification of the anticancer sequence for better efficacy and selectivity; (2) incorporation of a cancer-targeting sequence for tumor navigation. The sizes of the NC in water or in culture media containing serum were further monitored over one week. The NC size increased to ~170 nm in the presence of serum, but stabilized to ~100 nm during the incubation; while the size of NC in water was found to be stable during the first 3 days, whereas it formed large aggregates after 7 days of incubation (Figure S3). It suggests that the stability of the NC should be improved for physiological conditions. (3) A cleavable polyethylene glycol (PEG) shield could be incorporated to improve the stability and bioavailability while allowing the contact between peptides and tumor cells upon cleavage. Our recently accepted work discussed how environmental stimuli and surface chemistry influence the activity of cationic PAs [48]. These factors could be considered in the design of cationic PAs for further improvements. 

## 4. Materials and Methods

### 4.1. Materials

The peptide PAH6 (Pal-H_6_G_3_KLAKLAKKLAKLAK-NH_2_, MW 2755.43) was purchased from Canpeptide Inc. (Montreal, Québec, Canada) with the purity >95% (see Figure S4). Briefly, the peptide synthesis followed a solid-phase peptide synthesis method on Rink amide MBHA resin (AAPPTec, Louisville, KY, USA) manually with Fmoc (fluorenylmethyloxycarbonyl) chemistry. The deprotection of Fmoc (AAPPTec, Louisville, KY, USA) was conducted by the treatment with 20% piperidine (Sigma, Mississauga, ON, Canada) in DMF (*N*,*N*-dimethylformamide) (Sigma, Mississauga, ON, Canada), followed by coupling with the activated carboxyl group of the next amino acid. The coupling process was monitored with the Kaiser test. The synthesized peptide was cleaved from the resin with 95% TFA (trifluroacetic acid) (Sigma, Mississauga, ON, Canada). The purification of the peptide was performed using a Waters LC200 preparative system (Waters, Milford, MA, USA) equipped with a Phenomenex Gemini C18 column (Phenomenex, Torrance, CA, USA). Doxorubicin was purchased from LC laboratories (Woburn, MA, USA). The ATP-aptamer and its complementary DNA strand (sequences are listed in Table S1) were purchased from ThermoFisher Scientific (Mississauga, ON, Canada). All the other chemicals were purchased from Sigma-Aldrich unless otherwise specified.

### 4.2. Preparation and Characterization of Dox-Loaded DNA and the Dox-DNA/PAH6 Nanocomplex

The peptide PAH6 was dissolved in Milli-Q water at the concentration of 5.5 mg/mL and sonicated for 0.5 h to make the PA fully dissolved in solution. This PAH6 solution was used as stock to prepare the samples for tests. The Dox was mixed with DNA scaffold at different mass ratios to prepare the Dox-loaded DNA. The intercalation of Dox into the DNA scaffold and Dox fluorescence recovery in the presence of ATP was monitored using a QM4-SE spectra fluorometer from Photon Technology International (PTI, London, ON, Canada). The fluorescence of Dox was excited at 495 nm and the emission spectra were collected from 550 to 750 nm. The Dox-DNA/PAH6 NC was prepared by mixing the PAH6 solution and the Dox-DNA solution at a 1:1 volume ratio, followed by a 30-s vortex. The particle size and zeta potential of the NC were measured by the Nano ZS Zetasizer (Malvern, UK).

### 4.3. Atomic Force Microscopy (AFM)

To visualize the morphology of the Dox-DNA/PAH6 NC, a 50 μL solution was dropped onto the freshly cleaved mica (SPI, West Chester, PA, USA) surface. After 15 min of incubation, the solution was discarded and the mica surface was washed twice with Milli-Q water. The dried mica was then used to acquire the AFM image at room temperature on a Dimension Icon AFM (Bruker, Billerica, MA, USA) under the peak force quantitative nanomechanical mapping (PF-QNM) mode.

### 4.4. In Vitro ATP-Triggered Dox Release

The Dox release was measured by adding 100 μL of the NC solution into a mini dialysis tube (Slide-A-Lyzer, Thermo Fisher, Mississauga, ON, Canada) against 1 mL of PBS buffer containing calcium and magnesium. The buffer solution was stirred at 200 rpm at 37 °C. The Dox fluorescence emission at 595 nm in the buffer solution, with or without the presence of ATP, was measured at predetermined time intervals to estimate the percentage of Dox released.

### 4.5. Monolayer Cell Culture

The A549 human nonsmall cell lung cancer cell line and NIH-3T3 mouse fibroblast cell line were purchased from the American Type Culture Collection (ATCC, Manassas, VA, USA). A549 cells were cultured in F12K media (HyClone, VWR, Mississauga, ON, Canada) containing 10% FBS and 1% penicillin-streptomycin (HyClone, VWR, Mississauga, ON, Canada). NIH-3T3 cells were cultured in DMEM media (HyClone, VWR, Mississauga, ON, Canada) containing 10% FBS (VWR, CA, USA) and 1% antibiotics. Cells were grown in flasks at 37 °C in a humidified atmosphere containing 5% CO_2_.

### 4.6. Cell Morphology Imaging

A549 human nonsmall cell lung cancer cells were seeded in a 96-well plate at a cell density of 5000 per well and incubated for 24 h. The cells were then treated with Dox (1 μg/mL), PAH6 (60 μg/mL), or Dox-DNA/PAH6 (11:60) containing 1 μg/mL of Dox. The cell morphologies were observed on an EVOS FL cell imaging system (Thermo Fisher, Mississauga, ON, Canada) after 60 min of incubation.

### 4.7. Cell Membrane Permeability Assay

The treated A549 cells as described in the cell morphology imaging step were washed twice with warm PBS, then incubated with PBS solution containing 300 nM DAPI (Thermo Fisher, Mississauga, ON, Canada) for 5 min. The DAPI solution was then removed and the cells were washed twice with warm PBS solution again. The steps follow the manufacturer’s manual. The cells were finally incubated with culture media and the fluorescence intensity of DAPI was measured on a FLUOstar plate reader (BGM, Ortenberg, Germany). 

### 4.8. Mitochondrial Membrane Potential Assay

A549 cells were seeded in a 24-well plate at a cell density of 60,000 per well and incubated for 24 h before treatment. The cells were then treated with Dox (2 μg/mL), PAH6 (120 μg/mL) or Dox-DNA/PAH6 (11:60) containing 2 μg/mL of Dox for 1 h. The cells were washed twice with warm PBS and then incubated with F12K media containing 300 nM MitoTracker Red (Thermo Fisher, USA) for 20 min under cell growth conditions. All the steps follow the recommendations from the manufacturer’s manual. After the staining step, the staining solution was removed and the cells were washed twice with prewarmed PBS solution and collected. The fluorescence intensity of MitoTracker Red was measured using a BD FACSCalibur flow cytometry (BD Bioscience, San Jose, CA, USA). The mean fluorescence intensity of nontreated cells was counted as 1 to normalize the fluorescence intensity of treated cells.

### 4.9. In Vitro Cytotoxicity Test

The cytotoxicity of Dox, PAH6, and Dox-DNA/PAH6 (11:40 and 11:60) against A549 cells and NIH-3T3 cells were determined using Cell counting kit-8 (CCK-8, Dojindo Molecular Technologies Inc., Rockville, MD, USA). A549 or NIH-3T3 cells were seeded in the 96-well plates at the cell density of 5000 per well. After 24 h of incubation, the growth media in each well was exchanged with 150 μL of fresh media. The cells were then incubated for 24 h after the addition of 50 μL of the samples. After the exchange of treatment with growth media, the CCK-8 solution was added into the media at a 1:10 volume ratio. The absorbance of each well at 450 nm was read after 1 h of incubation using a FLUOstar plate reader (BGM, Ortenberg, Germany). The readings from nontreated cells were used as the negative control, while the empty wells were used as background readings.

The IC50 values were analyzed using Prism 7 software (La Jolla, CA, USA) by fitting the data to a dose–response–inhibition equation. The synergistic effect was determined by the equation below, where CI stands for combination index. For each drug combination, the CI lower than 1 indicates drug synergy [49]. The CI values were calculated and listed in Table S2.

CI = (*IC50 of Dox in complex*)/(*IC50 of Dox alone*) + (*IC50 of PAH6 in complex*)/(*IC50 of PAH6 alone*)

### 4.10. 3D-Cultured A549 Spheroids and Anticancer Activity Test

To culture the cells on a 3D Hanging Drop Plate (3D biomatrix Inc, Ann Arbor, MI, USA), A549 cells were collected from the flask and resuspended in growth media at a density of 50,000 cells per mL. Then, 40 μL of the cell suspension was pipetted into the well of the 3D Hanging Drop Plate to form a hanging droplet. The cells were then incubated for 4 d under cell growth conditions, allowing the cells to form a solid and steady spheroid at the center of each hanging droplet. The bottom part of the 3D plate was filled with PBS solution to provide moisture. Subsequently, 10 μL of treatment solution was added into the hanging drop and the morphologies of treated spheroids were observed on an EVOS FL cell imaging system (Thermo Fisher, Mississauga, ON, Canada) over time.

For cytotoxicity study, the A549 spheroids were transferred into a 96-well plate with a U-shaped bottom by slowly adding 150 μL of media into the pendant drop. The 3D plate was then placed on the top of the 96-well plate and the liquid left in the 3D plate was subsequently transferred by centrifugation [50]. Treatment solutions were added to the 96-well plate and the cytotoxicity was determined using CCK-8 [51]. The readings from nontreated spheroids were used as the control for 100% viability.

## 5. Conclusions

In summary, we reported a NC comprised of a small-molecule anticancer drug Dox, an ATP-binding aptamer incorporated DNA scaffold, and a cationic PA, PAH6. The DNA scaffold is used to load Dox and provide a feature of ATP-triggered drug release. The Dox-loaded DNA and PAH6 formed a NC through electrostatic interactions. Utilizing the membrane disruptive ability of PAH6, the NC exhibited significantly enhanced therapeutic efficacy and a moderate selectivity towards cancerous cells, similar to PAH6. The NC also showed the ability to penetrate A549 spheroids. This work provides a new strategy for constructing nanomedicines using therapeutic agents to achieve features of high drug loading and therapeutic efficacy, improved therapeutic index, and tumor penetration.

## Supplementary Materials

Supplementary materials can be found at http://www.mdpi.com/1422-0067/19/9/2773/s1.
