# Supplementary material for: Therapeutic Peptide Amphiphile as a Drug Carrier with ATP-Triggered Release for Synergistic Effect, Improved Therapeutic Index, and Penetration of 3D Cancer Cell Spheroids
Source: Int J Mol Sci. 2018 Sep 14;19(9):2773. doi: 10.3390/ijms19092773 (PMC6165277; doi:10.3390/ijms19092773)
Supplement: Supplementary file 1 [file ijms-19-02773-s001.pdf]

Therapeutic Peptide Amphiphile as a Drug Carrier with ATP-triggered Release for Synergistic effect, Improved Therapeutic Index and Penetration of 3D Cancer Cell Spheroids

Sheng Lu [1,Δ], Feng Zhao [2,Δ], Qiuxin Zhang [2,3] and P. Chen [1,*]

[1] Department of Chemical Engineering and Waterloo Institute for Nanotechnology, University of Waterloo, Waterloo, Ontario, N2L 3G1, Canada; shanels825@gmail.com (S.L.)

[2] Faculty of Science, University of Waterloo, Waterloo, Ontario, N2L 3G1, Canada; q344zhang@edu.uwaterloo.ca (Q.Z.), zhaofeng87806698@163.com (F.Z.)

[3] College of Nano Science and Technology, Soochow University, Suzhou, Jiangsu Province 215123, China;

* Correspondence: p4chen@uwaterloo.ca; Tel.: +1-519-888-4567, ext. 35586

Δ The authors contribute equally to this work

Supporting information

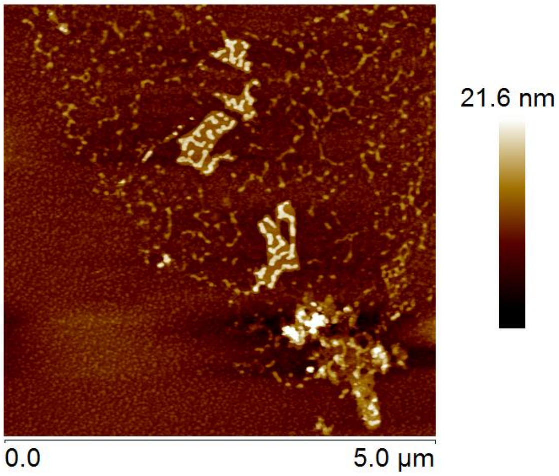

Figure S1. AFM image of PAH6 at the concentration of 60 µg/ml.

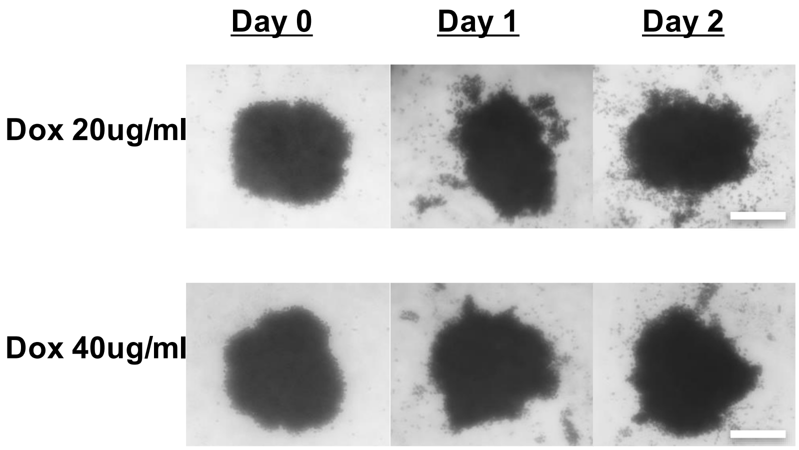

Figure S2. Optical micrographs of 3D A549 spheroids treated with Dox. The scale bar indicates 260 μm.

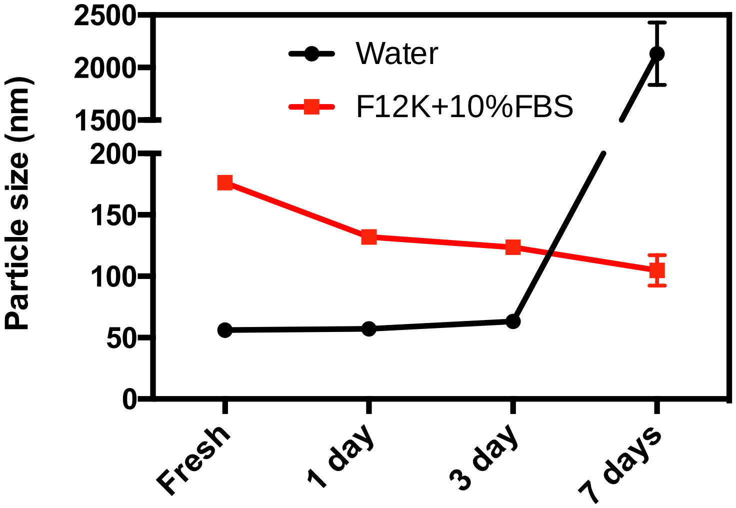

Figure S3. The averaged particle size of the Dox-DNA/PAH6 nano-complex in water or in F12K media containing 10% of FBS. The mass ratio of PAH6 to Dox-DNA is at 60:11. Error bars represent s.d. (n=3).

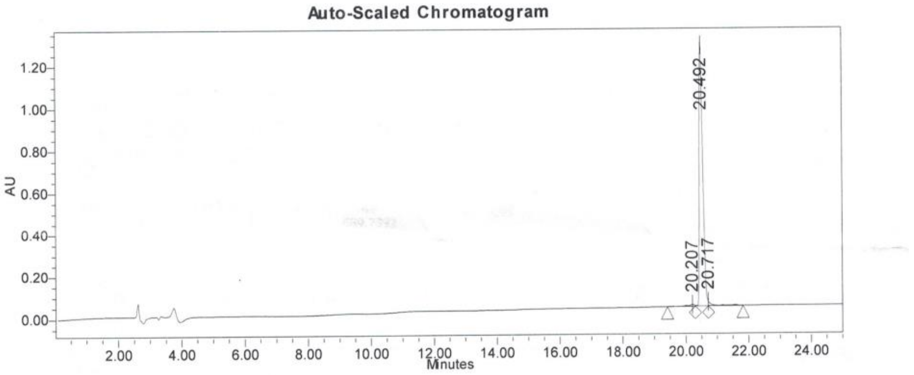

Peak Results

	Name	RT	Area	Height	% Area
1		20.207	95714	8677	0.96
2		20.492	9630995	1273264	96.67
3		20.717	236397	21648	2.37

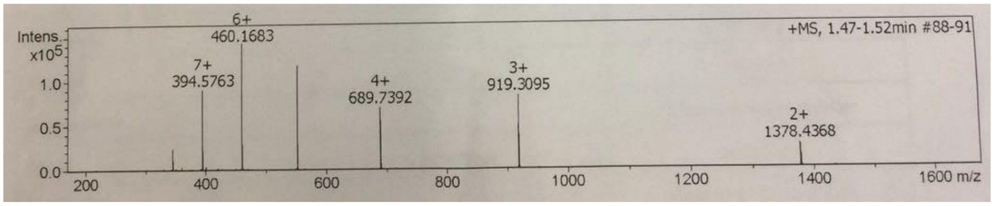

Figure S4. HPLC spectra of PAH6 (Top) and MS results for PAH6 (bottom) provided by the supplier.

Table S1. The sequences of oligonucleotides.

Oligonucleotide	Sequence
**ATP aptamer**	5'-ACC TGG GGG AGT ATT GCG GAG GAA GGT-3'
**Complementary DNA of ATP aptamer**	5'-ACC TTC CTC CGC AAT ACT CCC CCA GGT-3'

Table S2. Combination index values of Dox-DNA/PAH6 on different cell lines.

CI values	A549	HCT116	NIH-3T3
Dox-DNA/PAH6 (11:40)	**0.72**	**N/A**	**>1**
Dox-DNA/PAH6 (11:60)	**0.43**	**0.51**	**>1**
